# Caribbean multi-centre study of *Klebsiella pneumoniae*: whole-genome sequencing, antimicrobial resistance and virulence factors

**DOI:** 10.1099/mgen.0.000266

**Published:** 2019-04-29

**Authors:** Eva Heinz, Richard Brindle, Andrina Morgan-McCalla, Keisha Peters, Nicholas R. Thomson

**Affiliations:** ^1^​ Wellcome Trust Sanger Institute, Hinxton, UK; ^2^​ Liverpool School of Tropical Medicine, Liverpool, UK; ^3^​ Caribbean Public Health Agency, Port of Spain, Trinidad and Tobago; ^4^​ University of Bristol, Bristol, UK; ^5^​ University of the West Indies, Mona, Jamaica; ^6^​ London School of Hygiene and Tropical Medicine, London, UK

**Keywords:** *Klebsiella pneumoniae*, antimicrobial resistance, genomics

## Abstract

The surveillance of antimicrobial-resistant isolates has proven to be one of the most valuable tools to understand the global rise of multidrug-resistant bacterial pathogens. We report the first insights into the current situation in the Caribbean, where a pilot project to monitor antimicrobial resistance (AMR) through phenotypic resistance measurements combined with whole-genome sequencing was set up in collaboration with the Caribbean Public Health Agency (CARPHA). Our first study focused on *Klebsiella pneumoniae*, a highly relevant organism amongst the Gram-negative opportunistic pathogens worldwide causing hospital- and community-acquired infections. Our results show that not only carbapenem resistance, but also hypervirulent strains, are circulating in patients in the Caribbean. Our current data does not allow us to infer their prevalence in the population. We argue for the urgent need to further support AMR surveillance and stewardship in this almost uncharted territory, which can make a significant impact on the reduction of antimicrobial usage. This article contains data hosted by Microreact (https://microreact.org).

## Data Summary

1. Raw sequencing data have been deposited at the sequence read archive (SRA), and assemblies have been deposited at GenBank, accession numbers for all are given in Table S1.

2. The data of measured resistance phenotypes (VITEK) is provided in Table S1.

3. The tree file and associated metadata can be investigated and downloaded through the free online platform Microreact (https://microreact.org/project/S1-a7KAkV).

4. Additional tree files and alignments have been deposited in figshare, https://doi.org/10.6084/m9.figshare.7867760.v1.

**Significance as a BioResource to the community**

This BioResource contains the whole-genome sequence data of 270 *Klebsiella pneumoniae* isolates, information about encoded resistance genes and the phylogeny of the isolates, their distribution in the global *K. pneumoniae* population and their resistance phenotype data as determined by the VITEK 2 compact system. The isolates are recent (2017 through 2018) and represent clinically relevant patient isolates from 15 different sites in 13 Caribbean states. These data will be of interest for researchers working on *K. pneumoniae* and other opportunistic pathogens, as well as those interested in mobile genetic elements carrying antimicrobial-resistance (AMR) cassettes. Our data is the only recent survey of antimicrobial-resistant opportunistic pathogens from multiple sites within the Caribbean, and is of high significance for the global surveillance of *K. pneumoniae* and AMR elements. This BioResource is made available through data provided with this article, as well deposition of the raw data in the relevant archives, and an interactive platform (Microreact) to enquire and download analyses (phylogenetic tree, metadata).

## Introduction

The increasing level of antimicrobial resistance (AMR) in bacterial pathogens is one of the biggest worldwide threats for public health [1]. The spread is amplified as mobile resistance elements can cross both geographical and species borders, and the *Enterobacteriaceae* are especially prone to disseminating plasmids encoding AMR genes [2]. Monitoring the spread of resistant strains and resistance elements is further complicated as most of these bacteria are opportunistic pathogens that can be carried asymptomatically as part of the human microbiota. The mobility of people today, thus, greatly contributes to their worldwide spread. The phenomenon has been recognized by the major public-health agencies, and several surveillance programmes have been set in place to assess the prevalence of AMR in bacteria. This facilitates more informed decisions for interventions, guidelines for AMR practice and contributes to our understanding of the mechanisms leading to dissemination of AMR and the emergence of new resistances or high-risk lineages [1].

**Fig. 1.**
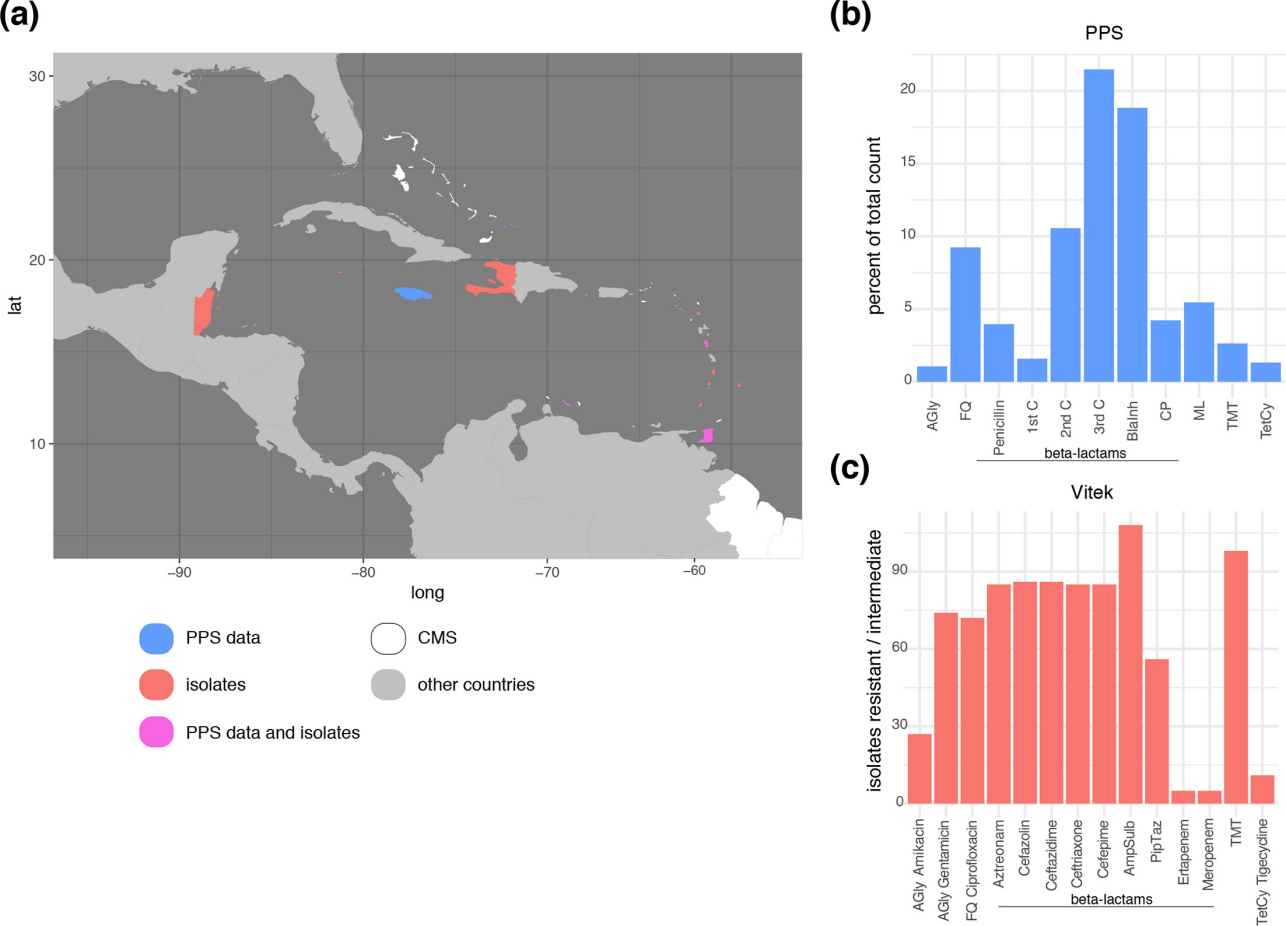
Comparing PPS data with phenotypic resistance profiles of the bacterial isolates. (a) Map showing the CMS (white), highlighting the countries contributing data to the PPS (blue), contributing bacterial isolates (red) and contributing to both (magenta). (b) Percentage of the total prescribed antibiotics during the PPS grouped into the main classes. AGly, aminoglycosides; FQ, fluoroquinolones; ML, macrolides; TMT, trimethoprim-sulfobactam; TetCy, tetracyclines. β-Lactams are further split into penicillins, cephalosporins (C1, first generation; C2, second generation; C3, third generation), β-lactam-β-lactamase-inhibitor combinations (BlaInh) and carbapenems (CP). (c) Phenotypic resistance data from the VITEK screening, showing the total number of strains, the main antimicrobial classes are indicated as in (b).

The Caribbean is a setting with a highly mobile population. The Caribbean Public Health Agency (CARPHA) incorporates 21 island states and 3 located in the Central and South American mainland (http://carpha.org/Who-We-Are/Member-States; Fig. 1a). This project was launched as part of a longitudinal AMR surveillance strategy in the Caribbean, initiated with funding from the United States Centers for Disease Control and Prevention (CDC) in 2016, to provide insight into the current state of AMR and to develop an antimicrobial stewardship programme. AMR surveillance is essential to identify potentially problematic clones and resistances, prevent future epidemics and recognize on-going epidemics, set measures to prevent further spread of high-risk clones, and better inform antimicrobial usage for health-care workers. To be effective, AMR surveillance needs to be established in combination with infection control and antimicrobial stewardship.

Our pilot project targeted *Klebsiella pneumoniae*, a member of the *Enterobacteriaceae* and recognized as one of the greatest threats for public health amongst multi-resistant Gram-negative opportunistic pathogens [3, 4]. A Caribbean-wide point prevalence survey (PPS) showed high usage of β-lactam antibiotics, especially third-generation cephalosporins, as well as quinolones, macrolides and a considerable degree of carbapenem usage (Figs 1b and S1, available with the online version of this article). We report the results of the first two surveys of isolates collected across the CARPHA member states (CMS). The first batch of isolates were collected in early 2017 with a second set of samples submitted to CARPHA during first half of 2018. Unfortunately, funding for this project has ceased along with CARPHA-based AMR surveillance. We provide an important snapshot of AMR across the Caribbean, including phenotypic and genomic data, analysis of the virulence and antimicrobial determinants, and the phylogenetic distribution of the Caribbean isolates in the context of the global *K. pneumoniae* population structure [5].

## Methods

### Antimicrobial usage data

The point prevalence survey (PPS) data were collected, as part of the Caribbean antimicrobial stewardship training programme, between March and May 2018. A World Health Organization (WHO) 2017 draft data collection form was used as a template, which was modified by the pharmacists during the training programme to better suit the Caribbean island hospitals, and was sent to pharmacist teams, who collected the data. The sampling represents a single collection time per hospital (one or a few days depending on hospital size); an individual patient was sampled only once. Twelve hospitals from nine states (Anguilla, Antigua, Bermuda, Dominica, Grenada, Jamaica, Nevis, Trinidad, Turks and Caicos Islands) submitted data in time for analysis in June 2018. A total of 1248 patients were reviewed, of which 681 patients had been prescribed 1136 antibiotics.

### Sample collection

Isolates were submitted by hospitals from the CMS: Antigua, Barbados, Belize, Bermuda, Cayman Islands, Dominica, Grenada, Haiti, Saint Kitts, Saint Lucia, Saint Vincent and Trinidad. Contributing CMS were encoded to anonymise the hospitals and states. The isolates were not selected for submission in a formal or structured fashion, and submission was dependent on the availability of transport media and staff. The isolates were mainly from the bloodstream, wounds and urine samples, but also from a wide range of other sources, including cerebrospinal fluid (CSF); further details on the specimens, as well as all accession numbers and sequencing details, are given in Table S1. Phenotypes and antimicrobial susceptibilities were determined using the VITEK 2 compact system (bioMérieux) within the CARPHA Laboratory, Port of Spain, Trinidad.

### Sequencing and typing analyses

DNA was isolated using a QIAamp DNA mini kit, following manufacturer’s instructions, within the CARPHA Laboratory; Illumina sequencing libraries with a 450 bp insert size were prepared according to the manufacturer’s protocols and sequenced on an Illumina HiSeq2000 with paired-end reads with a length of 100 bp; accession numbers of all samples are given in Table S1. The data was *de novo* assembled using the pipeline as described by Page *et al*. [6], and annotated with Prokka v1.5 using the -genus *Klebsiella* option [7]. Multiple locus sequence types (STs) were predicted as described previously [8]. Capsule (K-) and O-antigen (O-) types were predicted using Kaptive [9] [scripts and databases were retrieved from Kaptive (https://github.com/katholt/Kaptive, downloaded 15. 04. 2018)].

### AMR and virulence prediction

The presence of AMR genes and plasmid replicons was predicted using the srst2-argannot version as available for ariba [10–12] and plasmid replicons [13], respectively. *Klebsiella*-specific virulence determinants were investigated using Kleborate (https://github.com/katholt/Kleborate), which also provides known SNP-based resistance determinants for fluoroquinolone resistance (*gyrA*, *parC*) and colistin (none detected in this study). Boxplots and testing for significant differences between AMR determinants per ST were calculated using the ggplot2 boxplot function with the stats_compare_means function from the ggpubr package with the standard settings comparing indicated pairwise groups as well as a global comparison (https://rpkgs.datanovia.com/ggpubr/index.html, http://www.sthda.com/english/rpkgs/ggpubr) [14].

### Pan-genome and core gene analyses

The pan-genome was determined and core gene alignments were generated using Roary v3.7.0 [15] with the default conditions using mafft v7.205 [16]; SNPs were first extracted using snp-sites v2.3.2 using only ATGC columns (-c) [17], and then a maximum-likelihood tree was calculated with RAxML v8.2.8 with the general time-reversible (GTR) substitution matrix and gamma model of rate heterogeneity (-m GTRGAMMA), the rapid hill-climbing tree search (-f d) and 100 bootstrap replicates for support values [18]. The data were visualized with the ggtree and ggplot2 packages in R [14, 19]. As some of the publicly available strains were only available as reads for the core gene analysis of ST86, all isolate data used in the analyses was assembled using Shovill v1.0.1 (https://github.com/tseemann/shovill) with Spades as implemented and an expected genome size of 5.8 Mb, and annotated using Prokka as above. The pan-genomes of these smaller selections were generated using Roary as described above but disabling paralogue splitting (-s).

### Core genome phylogenies

The mapping and core genome alignment preparation was performed using snippy v4.3.3 (https://github.com/tseemann/snippy) with reads if available or otherwise with the snippy -contigs input option to shred assemblies. Recombination was removed with gubbins v1.4.10 ([20]; FastML 2 with Jukes Cantor model) after removing special characters with the snippy-clean_full_aln script provided with Snippy, and ACGT-only SNPs were extracted from the recombination-free alignment using snp-sites with the -c option as above. The resulting alignment was used for tree calculation with iqtree v1.6.5 [21] with the GTR model and gamma correction using ASC (ascertainment bias correction) for SNPs-only alignments (-m GTR+G+ASC) and 100 bootstrap replicates (-bb 100). Pairwise SNP distances were calculated using the dist.gene() function from the ape package for R [22], and visualized using ggplot2, ggtree and gheatmap [14, 19]. The mapping for the main STs (11, 15, 307, 405) was performed against closed reference genomes of the same or related STs [ST11, CP025951.1; ST15, CP008929.1; ST307, NCTN01000001 (not a closed chromosome, but representing an >5 kb contig); ST405, CP008929.1], and an outgroup from closely related STs was included (for ST11, AMR0203; ST15, AMR0417; ST307, AMR0163 and ERS2489012; ST405, AMR0445 and CP008929.1 used as a reference but also removed for the visualization as no ST405 reference was available). The consensus trees retrieved from iqtree were rooted on the outgroup lineages in FigTree (http://tree.bio.ed.ac.uk/software/figtree/); for visualization only, the outgroup branch was removed after using it to root the tree. The original alignments and trees can all be found on figshare (https://doi.org/10.6084/m9.figshare.7867760.v1). The mapping and pairwise SNP distances for *Klebsiella quasipneumoniae* subsp. *quasipneumoniae*, *K. pneumoniae sensu stricto* and ST86 were calculated as described above, using CP029597.1, AP006725.1 and CP006648.1 as references, respectively, and no outgroups were included.

## Results

### Antimicrobial usage in the region

A first initiative included the PPS of antimicrobial usage in the region. As this represents the pilot of setting up surveillance in the region and relied on voluntarily submitted data and capacity, the hospitals providing data for the PPS do not fully overlap with the hospitals providing the isolates described below, but cover the catchment area and give a broad overview of local usage (Fig. 1a). The PPS shows that β-lactams were by far the most used intravenous antimicrobials (ceftriaxone 22.5 %, amoxicillin-clavulanic acid 13.3 %, cefuroxime 10.4 %, piperacillin-tazobactam 7.6 %) with the exception of comparable high usage of metronidazole (17.8 %; Figs 1b and S1). Quinolones and macrolides were the predominant orally prescribed antimicrobials (20.3 and 21 %, respectively), as well as high levels of amoxicillin-clavulanic acid and cefuroxime usage (13.8 and 11.2 %). In total, 60.6% of all used antimicrobials were β-lactams, with 4.2 % of the total carbapenems.

### Phenotypic description

The isolates included in this study were submitted by a total of 15 different hospitals in 12 CMS following two calls for isolates (Fig. 1a). Although the majority were isolated from urine or blood samples, others included isolates from wound infections, invasive isolates from abscesses and a CSF isolate from one fatal case of meningitis. A significant proportion of the isolates included in this study were resistant to fluoroquinolones, trimethoprim and β-lactams, which represent the major classes of antimicrobials used against Gram-negative infections (Fig. 1c). We note high carbapenem usage but low resistance in our isolates (Fig. 1b, c), and no usage or resistance could be seen for tigecycline (Figs 1 and S1). Phenotypic screening confirmed a high level of extended-spectrum β-lactamase (ESBL) presence (31.5 % resistant to ceftriaxone) and, to a similar extent, reduced susceptibility to other antimicrobial classes such as aminoglycosides (gentamicin 27 % resistant and 0.4 % intermediate) and fluoroquinolones (ciprofloxacin 26.7 % resistant); whereas only a small proportion (1.1 % resistant, 0.74 % intermediate) of the isolates were phenotypically carbapenem resistant. We also note a relatively low proportion of amikacin (0.74 % resistant, 9.3 % intermediate) and piperacillin-tazobactam (10 % resistant, 10.7 % intermediate) resistance, the latter representing a key alternative to carbapenems in treating ESBL-positive organisms (Fig. 1c).

### Sampled *K. pneumoniae* population structure

**Fig. 2.**
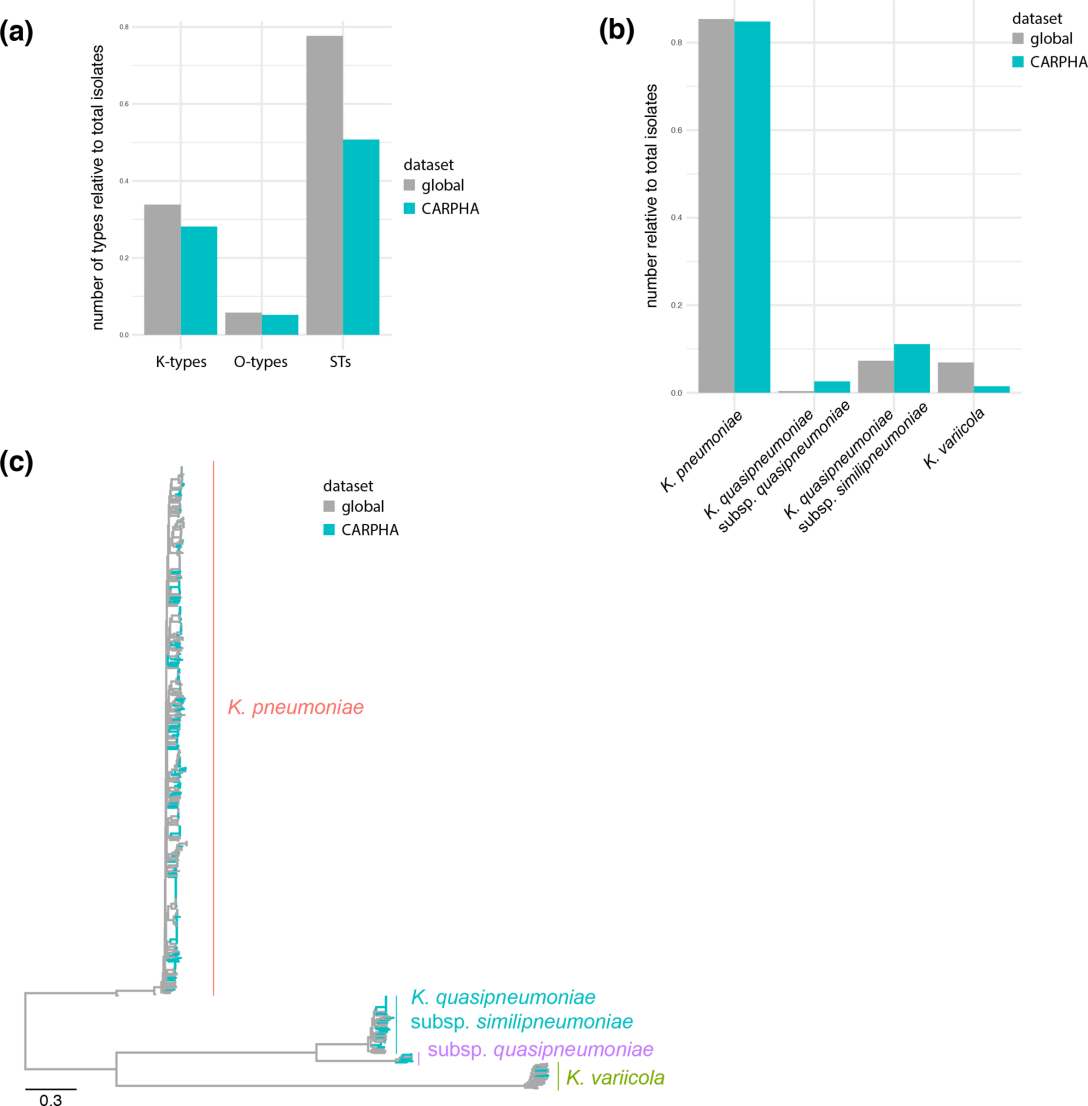
The Caribbean data in a global context. (a) Comparison of the diversity based on capsule (K), O-antigen (O) and STs compared with the global population study of Holt *et al.* from 2015 [5]. (b) Comparison of the three different species between the global collection and this study. (c) Phylogenetic analysis of the retrieved isolates demonstrates that they represent the global *K. pneumoniae* population as established by Holt *et al*. in 2015.

Using whole-genome sequences, the Caribbean isolates were compared to a global collection designed to capture the *K. pneumoniae* species complex population diversity [5]. The *K. pneumoniae* population in the Caribbean shows similar diversity when considering the O-antigen or capsule loci, as well as the range of multilocus STs present in this region (Fig. 2a), meaning that they are not comprised of only one or few widespread lineages in the Caribbean, but are representative of a genetically diverse established population. What is commonly summarized as *K. pneumoniae* represents a species complex, comprising *K. pneumoniae sensu stricto*, *K. quasipneumoniae* with its subspecies *quasipneumoniae* and *similipneumoniae*, and *Klebsiella variicola* [5, 23]. The latter was recently further classified as new subspecies were recognized (*variicola* and *tropicalensis*) [24], and an additional species, *Klebsiella africanensis*, was also identified only recently [24]. Our data shows a high diversity of isolates including three species of the sequence complex (*K. pneumoniae*, *K. quasipneumoniae*, *K. variicola*; Fig. 2b), and we note a high number of closely related members of *K. quasipneumoniae* subsp. similipneumoniae*.* Thirteen isolates of ST1605 were identified with no SNPs in the core genome compared to a reference genome of the subspecies (*K. quasipneumoniae* subsp. *similipneumoniae* strain ATCC 700603 CP029597.1 [25]; Fig. S2) and no clearly identifiable gene differences other than uncertainty through short-read sequencing, indicating one circulating lineage and possibly direct transmission events [26, 27]. *K. quasipneumoniae* subsp. *similipneumoniae* has recently been recognized as an important contributor to hospital infections [28–32]. We also notice higher numbers of *K. quasipneumoniae* subsp. *quasipneumoniae*, whereas *K. variicola* seems underrepresented, when compared to the broad *K. pneumoniae* diversity as established by Holt *et al.* in 2015 (Fig. 2c) [5].

### AMR determinants

**Fig. 3.**
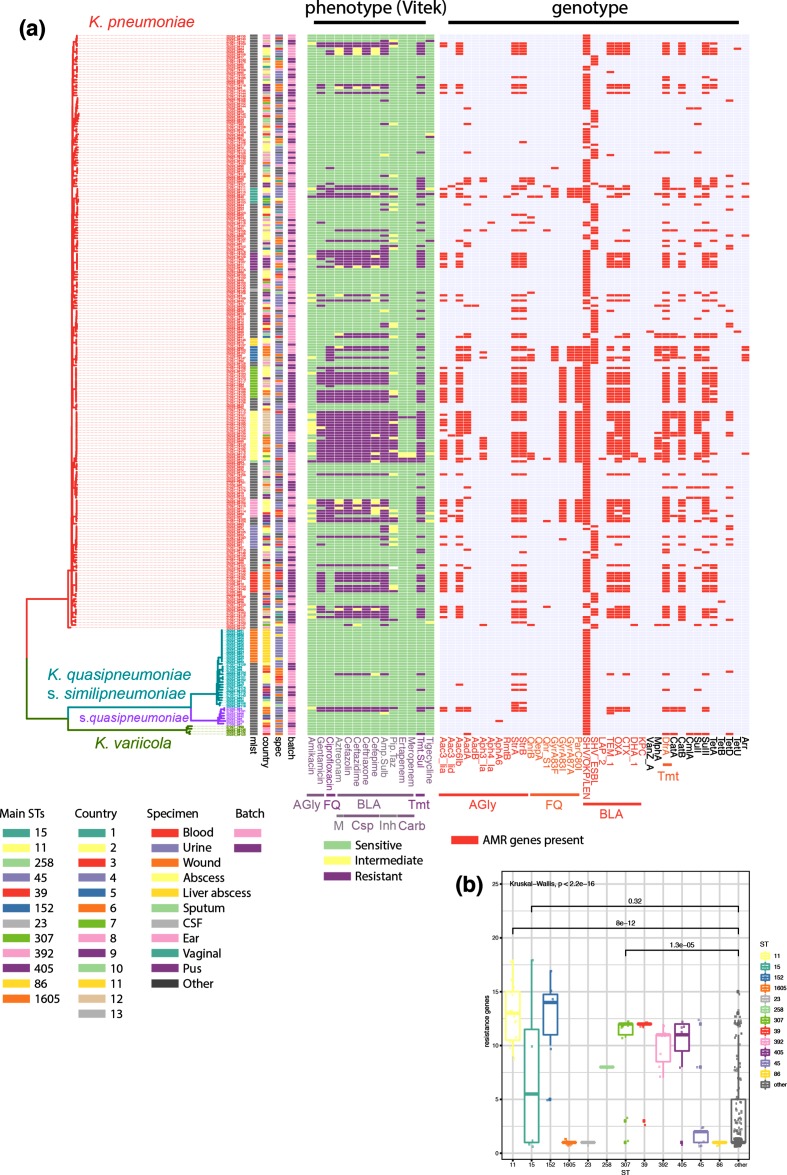
Whole-genome sequencing analysis reveals several high-risk clones with high AMR and hypervirulent lineages. The guidance tree is based on the core gene alignment as obtained by roary, and the colour strips represent, from the left, the major STs as determined by multilocus sequence typing (MLST), the country-code of isolation and the specimen from which the isolate was obtained, and whether these were delivered in the first or second batch (early/late 2017). The heat maps represent the measured resistance phenotype as determined by VITEK (green, sensitive; yellow, intermediate; violet, resistant), and the predicted resistance genes as well as chromosomal mutations known to confer resistance (gyrase and topoisomerase mutations conferring fluoroquinolone resistance). (b) Number of resistance determinants per strain, comparing the main STs with the background population. AGly, Aminoglycosides; FQ, fluoroquinolones; TMT, trimethoprim-sulfobactam.

There is little recent information available about how widespread AMR is in the region apart from single country reports [33–35], and no CMS are enrolled in the Global Antimicrobial Resistance Surveillance System (GLASS). However, the Caribbean is located between two hotspots of carbapenem-resistant *K. pneumoniae*, which are a recognized high risk in the USA (https://www.cdc.gov/hai/organisms/cre/index.html) and South America with levels of ESBL-mediated resistance over 80 % and carbapenem resistance over 25 % in several countries (https://www.paho.org/hq/dmdocuments/2017/2014-cha-informe-anual-relavra.pdf). Our analysis of the genomic data shows a high number of acquired-drug-resistance genes present in the genomes of a considerable number of isolates and STs (Figs 3a, b and S3), and a high diversity including ESBL genes (*bla*_CTX-M-14_, *bla*_CTX-M-15_, *bla*_SHV100_, *bla*_SHV101_, *bla*_SHV27_, *bla*_SHV38_, *bla*_SHV70_, *bla*_SHV98_, *bla*_SHV99_), AmpC-type β-lactamases (*bla*_DHA1_) and two carbapenemases (*bla*_KPC2_ and *bla*_KPC3_) (Fig. S3). The genotypic predictions of resistance largely match with their phenotypic resistance profiles; of 85 ceftriaxone-resistant strains, 83 encode an ESBL and/or carbapenemase (80 *bla*_CTX-M-15_, 1 *bla*_CTX-M-14_, 1 *bla*_CTX-M-15_ and *bla*_KPC2_, 1 *bla*_KPC3_) and of 3 carbapenem-resistant strains, 2 encode a carbapenemase (Fig. 3). The majority of observed resistant isolates, however, are clustered in several STs; including globally recognized high-risk clones ST11, ST15, ST307 and ST405 [4, 36] (Fig. 3b). Also present at low numbers, even in our limited number of samples, are high-risk clones such as ST258 [37]; although this isolate did not carry a carbapenemase gene. To gain further insights into the high-risk STs and the level of diversity amongst isolates of the same ST, we performed several high-resolution analyses within these and in context with publicly available data.

### Major antimicrobial STs

**Fig. 4.**
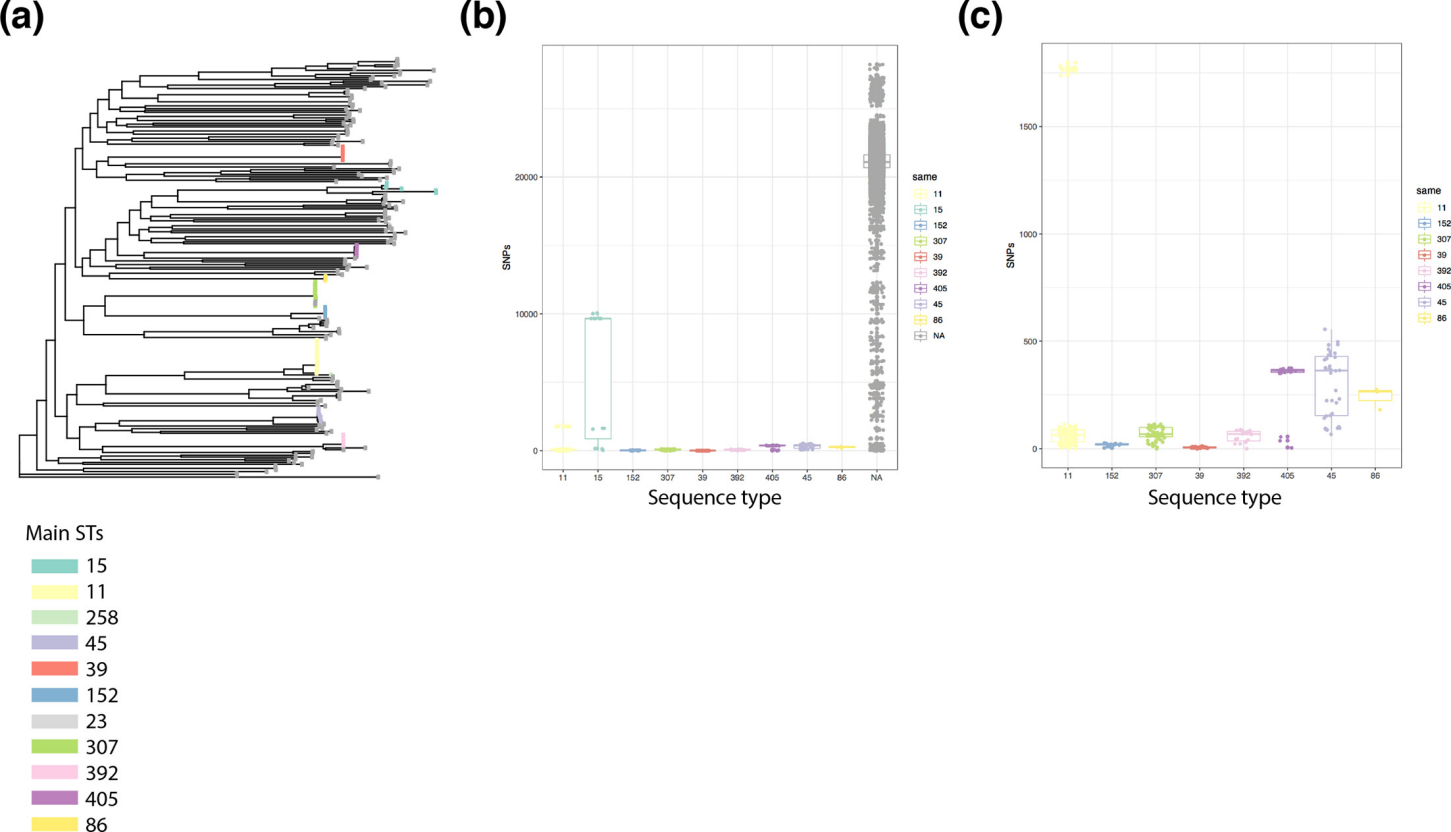
The diversity of *K. pneumoniae sensu stricto* isolates. (a) Phylogenetic tree based on the core genome alignment, mapping all *K. pneumoniae sensu stricto* strains from this study (reference used: AP006725.1) and removing recombination. The tips are coloured according to the main STs as indicated in the legend or shown in dark grey for all other STs. (b) Pairwise SNP comparison, grouping within-ST pairs for the main STs, and others (within less prevalent STs and not within STs). (c) Subset of (b) showing only the main STs to give a higher resolution in the low value range.

Our first experiment addressed the diversity of the different, at first sight seemingly clonal, STs. We, therefore, performed a core-genome analysis of all isolates belonging to *K. pneumoniae sensu stricto* against the same reference (AP006725.1), and compared the pairwise SNP distances (Fig. 4). The tree (Fig. 4a) clearly shows the deep branches between different STs, as reflected in the SNP distances when comparing the pairwise distances within the main STs to all other pairwise hits (Fig. 4b). We also observed differences within the main STs, where ST15 shows high and ST11 visible diversity on this very large scale (Fig. 4b), focusing on the smaller scale highlights that even within the seemingly clonal STs, clear differences can be observed (Fig. 4c).

**Fig. 5.**
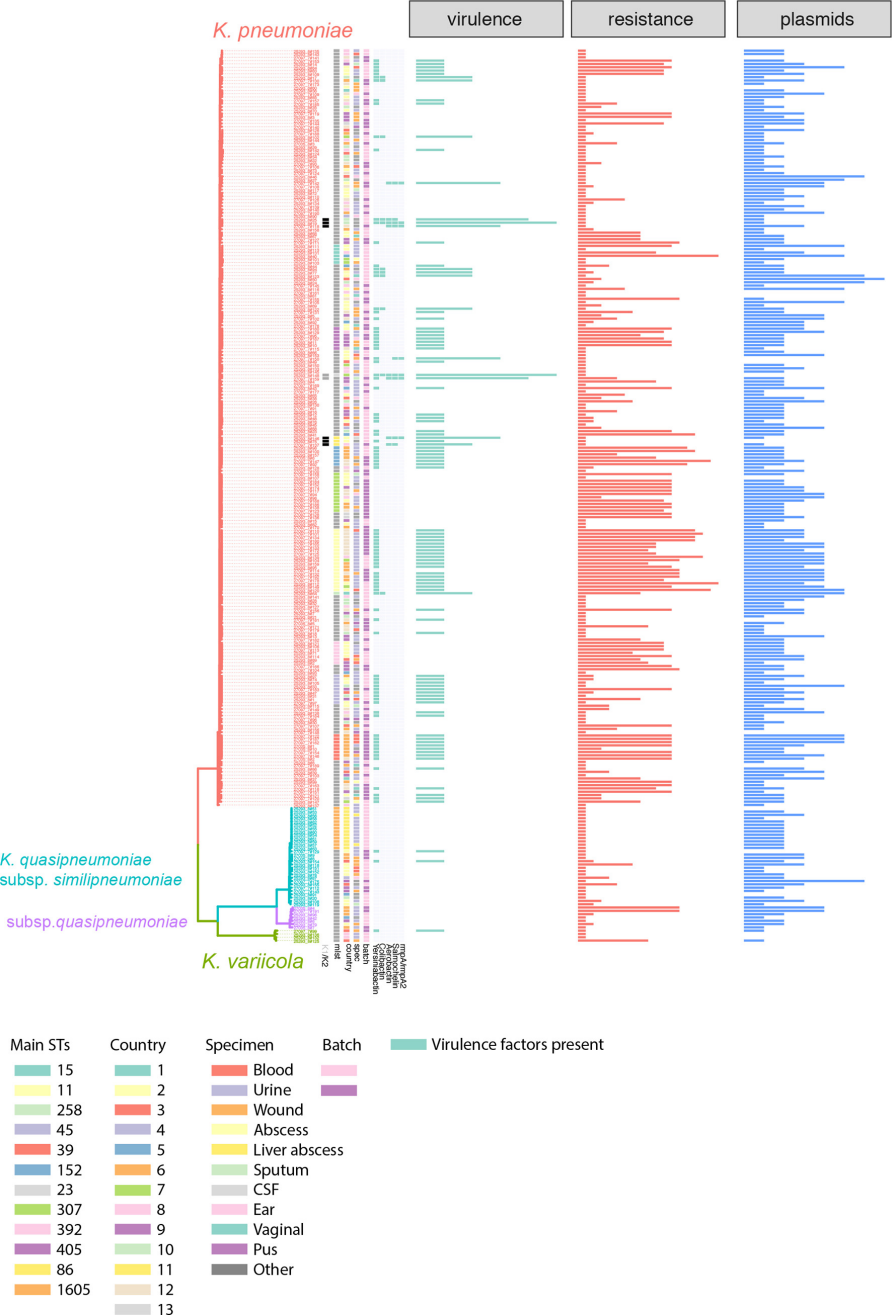
Virulence factors and their distribution compared to AMR and plasmid replicons. Guidance tree as in Fig. 3; the virulence determinants are shown as predicted by Kleborate. The panels show the summarized number of virulence determinants, AMR genes and plasmid replicons (left to right) as indicated. Details of all AMR alleles and plasmid replicon types are given in Fig. S3 and in the Microreact files.

To get insights into the diversity within these STs beyond their AMR and virulence determinants, we performed core genome SNP analyses, using a reference sequence of the same or related STs to increase coverage of the highly diverse *Klebsiella* genome (Fig. S4, Table S2; details in Methods). We furthermore included published data from several studies, adding both data from local outbreaks, as well as longitudinal surveillance or sporadic isolates unrelated to the related outbreaks, to bring our isolates into context of clonal lineages as well as circulating diversity [5, 32, 37–54]. This matches the varying patterns of AMR determinants we see (Figs 3, S3 and S4), with ST15, ST307 and ST405 representing several clearly distinct, diverse lineages circulating in the Caribbean, whereas ST11 is, with one exception, a monophyletic group (Fig. S4). The comparison with included data from local outbreaks (ST15 outbreak in Nepal [39], ST405 in Spain [48], ST11 in PR China [55]) clearly indicates a high diversity circulating; with occasional likely direct transmission networks within the area or hospitals leading to small groups of almost-identical isolates (Fig. S4). This is also indicated by the SNP distances; whereas the isolates from this study differ by >100 SNPs in ST15 and ST405 and do not branch as one monophyletic lineage, ST11 and ST307 isolates differ by <10 SNPs and branch off as monophyletic lineages (Fig. S4). Although this of course depends largely on the available data, the close SNP distances seem to indicate a transmission network within the island region that is likely underestimated given our incomplete sampling.

### (Hyper)virulence factors

*K. pneumoniae* is increasingly recognized as causing severe, community-acquired invasive disease including liver abscess, pneumonia or meningitis, which are commonly associated with different clonal groups than the highly drug-resistant isolates [56]. The main factors associated with hypervirulence are the hypermucoid phenotype through the capsule regulator gene *rmpA/rmpA2*, the siderophore aerobactin, and the capsule types K1 or K2, with the two former encoded on a virulence plasmid characteristic for invasive strains [56] (Fig. 5). In addition to the main high-risk clones with respect to AMR, we noticed isolates belonging to lineages known to harbour hypervirulent isolates [56]: one ST23 isolate (AMR0157; urine isolate), two isolates belonging to ST65 (AMR0288 and AMR0296; unknown isolation source) and three to ST86 (17–02612, AMR0062, AMR0879; CSF, respiratory, urine isolates; Figs 3 and 5) [57–61]. Additional virulence factors are further siderophore systems, encoding salmochelin, yersiniabactin and colibactin. The ST86 isolate 17–02612 was derived from fatal case of community-acquired meningitis, which affected a previously healthy patient. A comparison of our isolates with other publicly available ST86 isolates included a meningitis case in geographical proximity ([61]; Fig. S5). Whilst these two meningitis cases are both typed as ST86, it seems unlikely they were derived from one circulating lineage as they show 405 SNPs difference whilst having been isolated only 2 years apart (Fig. S5). However, we notice that the meningitis cases encode the full potential of virulence factors associated with hypervirulent *K. pneumoniae*, importantly the hypermucoidy regulator *rmpA/rmpA2*, as well as aerobactin and salmochelin (Figs 5 and S5). All ST86 isolates encode capsule type K2, which is often associated with invasive *K. pneumoniae* isolates (Figs 5 and S6). The ST23 isolate and one of the ST65 isolates (AMR0288) also encode the genes for aerobactin, yersiniabactin, salmochelin and colibactin as well as the *rmpA* hypermucoidity regulator gene, indicating a diverse reservoir of hypervirulent strains circulating in the region (Fig. 5).

### Highly diverse pool of high-risk clones carrying resistance and virulence plasmids circulating in the Caribbean

**Fig. 6.**
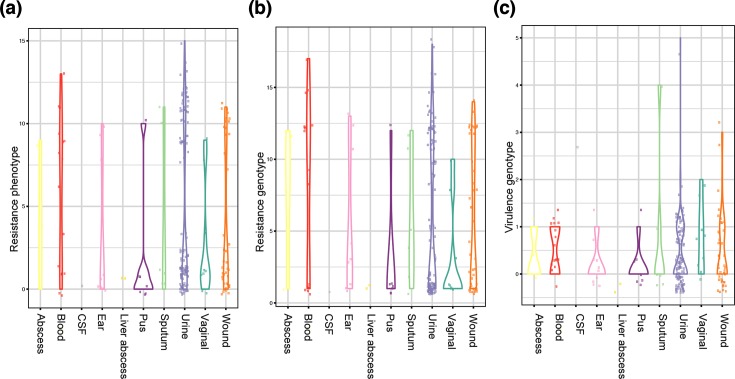
Bloodstream isolates are highly resistant but not enriched in virulence factors. Isolates are stratified by sample specimen, showing (a) the number of resistant or intermediate readouts on the VITEK system, (b) the number of predicted resistance determinants and (c) the number of predicted virulence determinants.

Comparing the number of resistance and virulence determinants shows the typical split distribution with highly virulent and highly resistant strains (Fig. 5), but the convergence between virulence and resistance cannot be observed in our limited sampling data, although all ingredients are present in the local gene pool. Whilst our sampling call was targeted at AMR surveillance and we, thus, see a high number of blood and urine isolates with high-resistance profiles but no (bloodstream isolates) or few (urine isolates) with an enriched number of virulence determinants (Fig. 6). The origins of strains with several virulence determinants includes sterile sites such as CSF and abscesses indicative of causing invasive disease as seen in the fatal meningitis case (Figs S5 and S6) [62].

## Discussion

This study aimed to establish a genomic surveillance network across the Caribbean. Although this study was prematurely terminated, it has provided important data. We show that several high-risk multidrug-resistant bacterial clones are present in clinical samples collected across the Caribbean. For *K. pneumoniae*, these include STs ST258, ST11, ST15, ST307 and ST405. The diversity of the high-risk clones highlights that the risks from AMR are not limited to or described by the spread of a single high-risk lineage across different states or islands. Neither do we see indications for a single plasmid or genetic island moving between relevant pathogens causing disease, but a large pool of diverse *K. pneumoniae* lineages and resistance genes distributed across the region.

Given the limitations and the lack of a structured surveillance framework, we cannot conclude whether our data accurately reflects the true prevalence or the full extent of spread of these bacteria across this region. However, even given our limited sampling, there is a significant risk of rapid spread or ongoing, unnoticed epidemics of some of these high-risk clones, the presence and circulation of which will be hidden from view without using highly accurate approaches such as whole-genome sequencing.

Descriptions of infections caused by hypervirulent *K. pneumoniae* strains in the Caribbean are so far rare [61, 63], but given the lack of surveillance, these observations might only represent the tip of the iceberg. The presence of high-risk multidrug resistant and hypervirulent strains, most of which are carried on mobile elements, also bears the further threat of the convergence to a multidrug-resistant hypervirulent strain. This has been reported recently for ESBL-positive ST29 [64], KPC (*K. pneumoniae* carbapenemase)-positive ST11 acquiring hypervirulence features [47, 59], and hypervirulent ST23 acquiring AmpC DAC-1 and ESBL enzymes [65]. All these components are part of the *K. pneumoniae* pool circulating in the Caribbean. We argue that it is of crucial importance to continue the building of a systematic surveillance framework in the Caribbean, to fully assess the situation, provide informed guidelines for antimicrobial use and update these, as well as monitor high-risk clones and prevent outbreaks at their start.

## Supplementary Data

Supplementary File 1

Supplementary File 2
