# Supplementary material for: Caribbean multi-centre study of Klebsiella pneumoniae: whole-genome sequencing, antimicrobial resistance and virulence factors
Source: Microb Genom. 2019 Apr 29;5(5):e000266. doi: 10.1099/mgen.0.000266 (PMC6562249; doi:10.1099/mgen.0.000266)
Supplement: Supplementary File 1 [file mgen-5-266-s001.pdf]

# **Caribbean multi-centre study of *Klebsiella pneumoniae*: whole genome sequencing, antimicrobial resistance and virulence factors.**

Eva Heinz[1,2*], Richard Brindle[3,4], Andrina Morgan-McCalla[3,5], Keisha Peters[3], Nicholas R. Thomson[1,6]

1: Wellcome Trust Sanger Institute, Hinxton, UK

2: Liverpool School of Tropical Medicine, Liverpool, UK

3: Caribbean Public Health Agency, Port of Spain, Trinidad & Tobago

4: University of Bristol, Bristol, UK

5: University of the West Indies, Mona, Jamaica

6: London School of Hygiene and Tropical Medicine, London, UK

*Correspondence: eva.heinz@lstmed.ac.uk

**Supplementary data**

**Figure S1: Numbers of all orally (A) and peritoneal (B) prescribed antibiotics reported in the PPS.**

# Figure S1

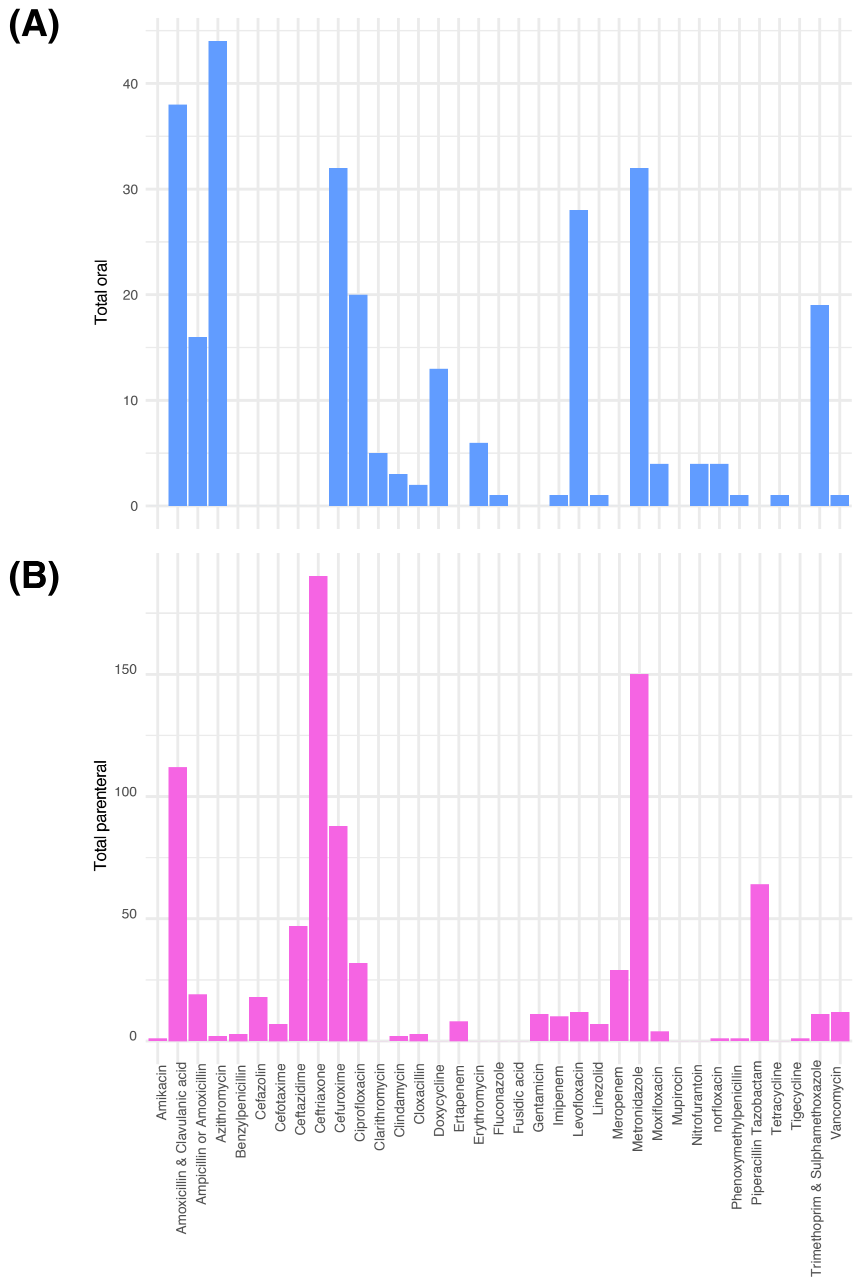

(A)
Total oral
0
10
20
30
40
(B)
Total parenteral
0
50
100
150
Amikacin
Amoxicillin & Clavulanic acid
Ampicillin or Amoxicillin
Azithromycin
Benzylpenicillin
Cefazolin
Cefotaxime
Ceftazidime
Ceftriaxone
Cefuroxime
Ciprofloxacin
Clarithromycin
Clindamycin
Cloxacillin
Doxycycline
Ertapenem
Erythromycin
Fluconazole
Fusidic acid
Gentamicin
Imipenem
Levofloxacin
Linezolid
Meropenem
Metronidazole
Moxifloxacin
Mupirocin
Nitrofurantoin
norfloxacin
Phenoxymethylpenicillin
Piperacillin Tazobactam
Tetracycline
Tigecycline
Trimethoprim & Sulphamethoxazole
Vancomycin

**Figure S2: Core genome analysis of *K. quasipneumoniae* subsp. *similipneumoniae* isolates.** Phylogenetic tree of the isolates (left) and corresponding pairwise SNP distances in a heatmap (right).

# Figure S2

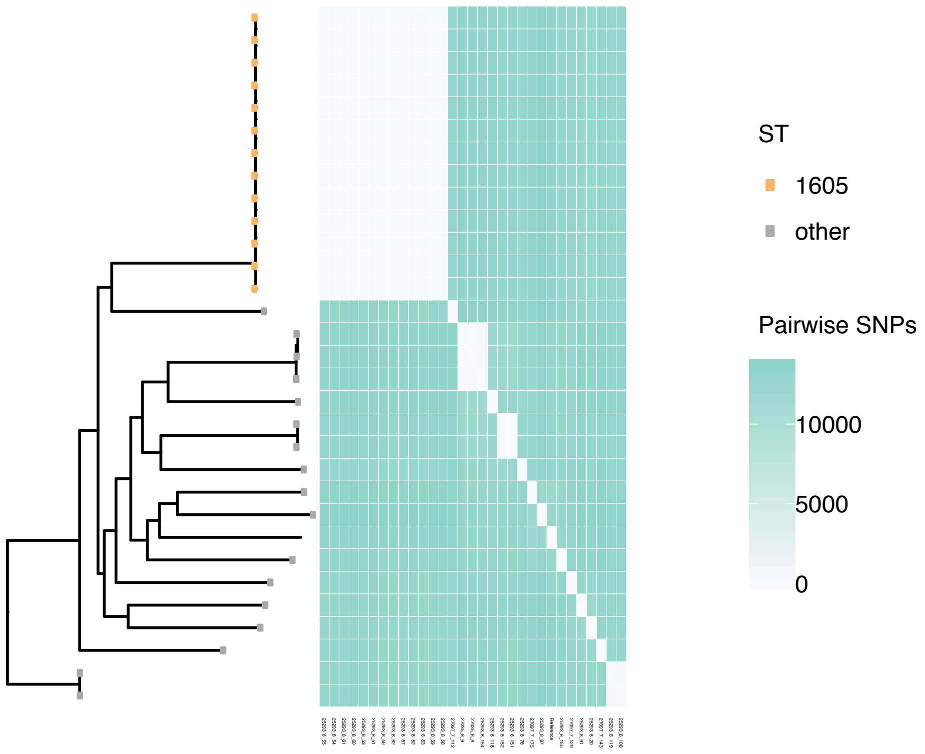

**Figure S3: Antimicrobial resistance gene alleles for all analysed genomes in this study.**

# Figure S3

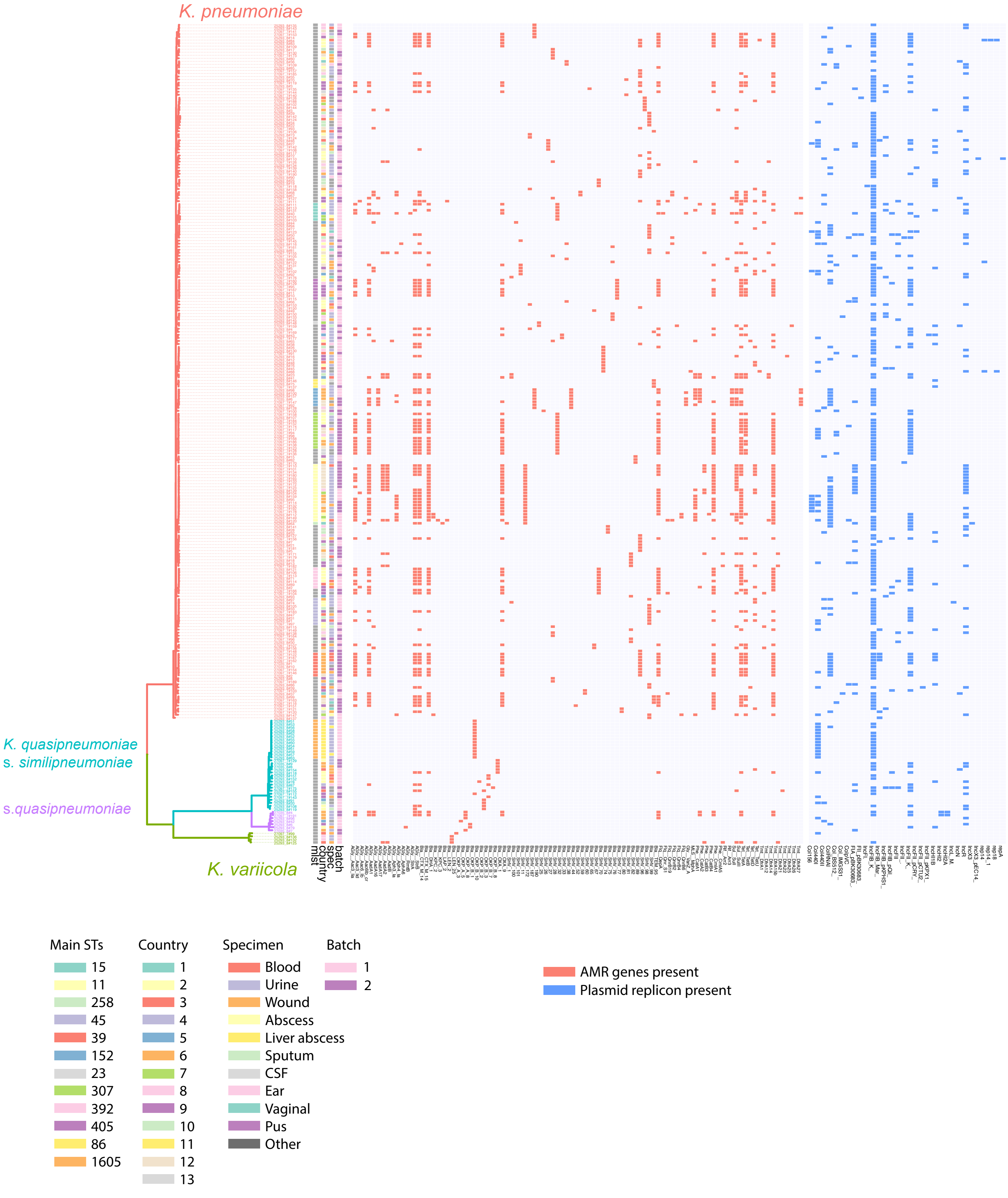
K. pneumoniae
K. quasipneumoniae
s. similipneumoniae
s.quasipneumoniae
K. variicola
mlst
country
spec
batch
Main STs
15
11
258
45
39
152
23
307
392
405
86
1605
Country
1
2
3
4
5
6
7
8
9
10
11
12
13
Specimen
Blood
Urine
Wound
Abscess
Liver abscess
Sputum
CSF
Ear
Vaginal
Pus
Other
Batch
1
2
AMR genes present
Plasmid replicon present

**Figure S4: Core genome analysis for main antimicrobial resistant, globally spread STs.** Mapping tree (left) in the context of public data and the corresponding pairwise SNP distances after removing recombination shown as heatmap (right) for **(A)** ST11**, (B)** ST15**, (C)** ST307**, (D)** ST405**.** The published strains included in the analyses are given in Dataset S2.

# Figure S4

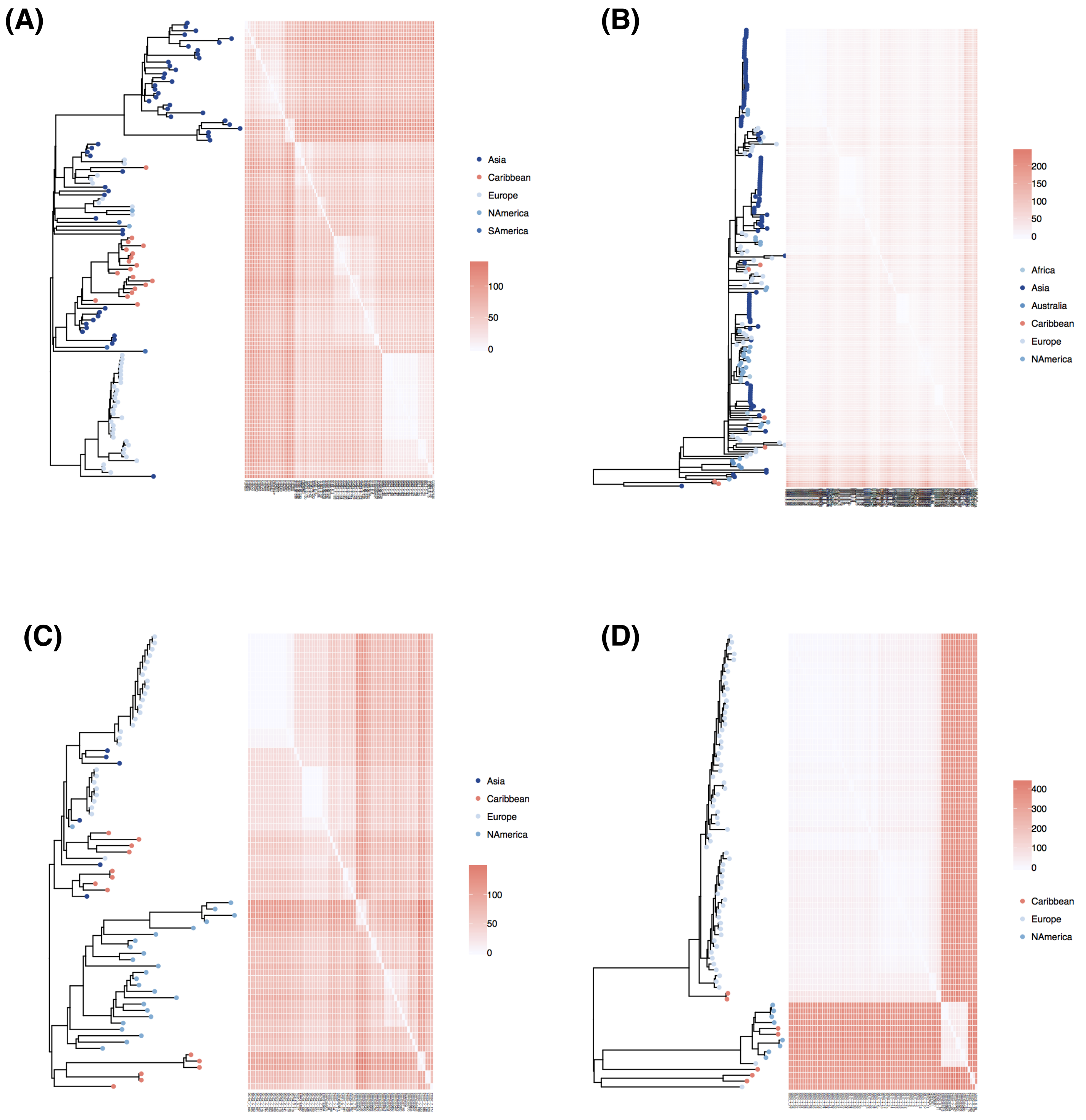
(A)
Asia
Caribbean
Europe
NAmerica
SAmerica
100
50
0
(B)
200
150
100
50
0
Africa
Asia
Australia
Caribbean
Europe
NAmerica
(C)
Asia
Caribbean
Europe
NAmerica
100
50
0
(D)
400
300
200
100
0
Caribbean
Europe
NAmerica

**Figure S5: Comparison of two meningitis cases in the Caribbean.** The mapping tree shows the two cases ([61], this study) in the context of other isolates. The core genome tree alignment was used to calculate pairwise SNP distances as in Figure S4 (middle panel), and the presence or absence of *K. pneumoniae*-specific virulence determinants associated with invasive disease [5] is shown on the right panel.

# Figure S5

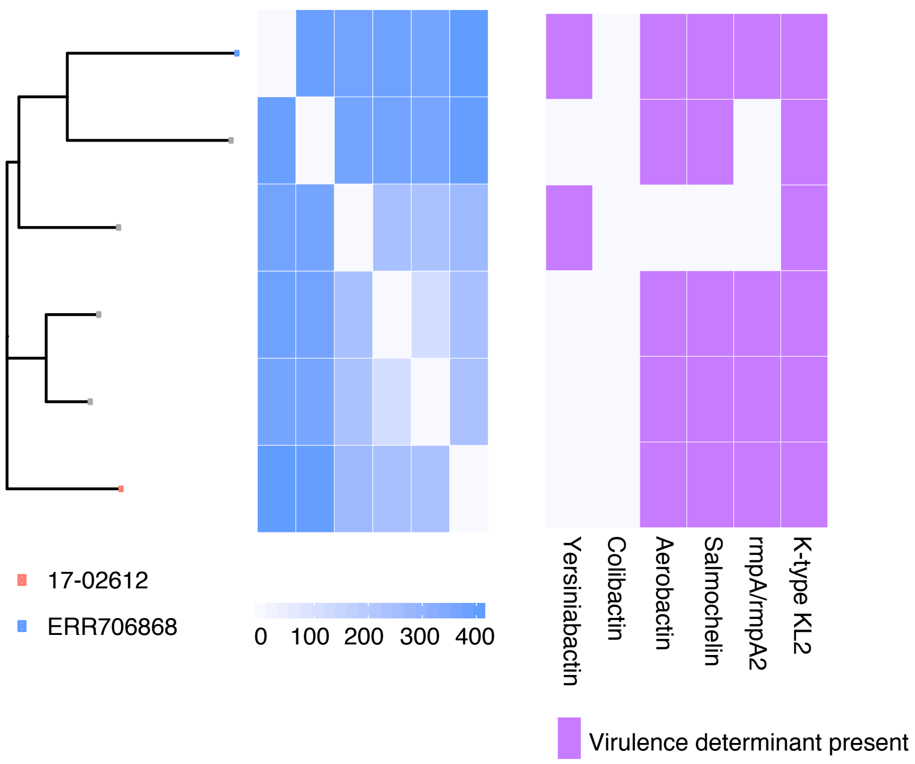

17-02612
ERR706868
0
100
200
300
400
Yersiniabactin
Colibactin
Aerobactin
Salmochelin
rmpA/rmpA2
K-type KL2
Virulence determinant present

**Figure S6: Capsule (K-) and LPS O-antigen (O-type) genotype distribution in all isolates.**

# Figure S6

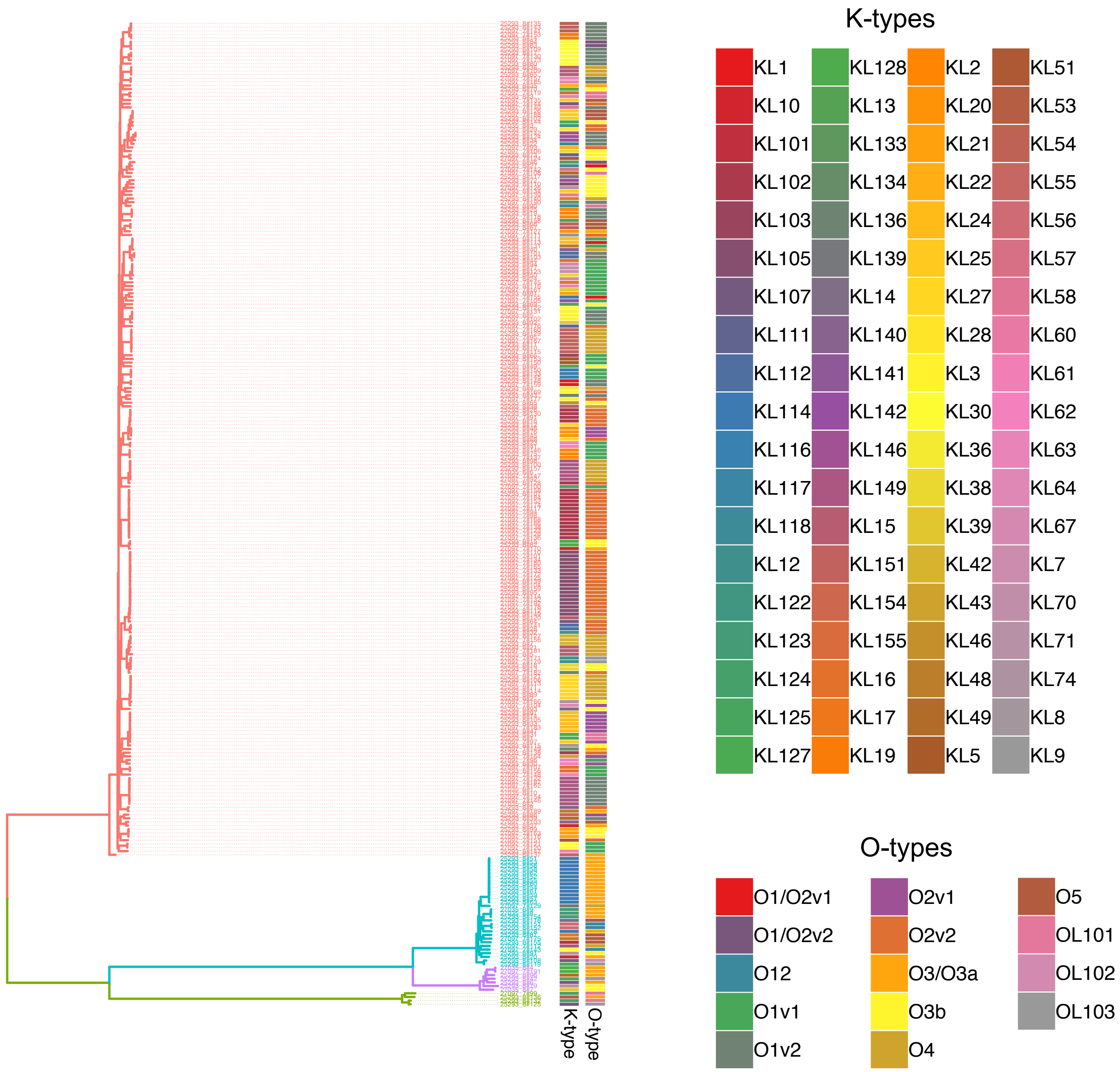
K-type
O-type
K-types
KL1
KL10
KL101
KL102
KL103
KL105
KL107
KL111
KL112
KL114
KL116
KL117
KL118
KL12
KL122
KL123
KL124
KL125
KL127
KL128
KL13
KL133
KL134
KL136
KL139
KL14
KL140
KL141
KL142
KL146
KL149
KL15
KL151
KL154
KL155
KL16
KL17
KL19
KL2
KL20
KL21
KL22
KL24
KL25
KL27
KL28
KL3
KL30
KL36
KL38
KL39
KL42
KL43
KL46
KL48
KL49
KL5
KL51
KL53
KL54
KL55
KL56
KL57
KL58
KL60
KL61
KL62
KL63
KL64
KL67
KL7
KL70
KL71
KL74
KL8
KL9
O-types
O1/O2v1
O1/O2v2
O12
O1v1
O1v2
O2v1
O2v2
O3/O3a
O3b
O4
O5
OL101
OL102
OL103
